# Analysis of panel physician inquiries to U.S. TB Centers of Excellence, 2018–2022

**DOI:** 10.5588/ijtldopen.24.0056

**Published:** 2024-11-01

**Authors:** E. Leithead, S. Subramanian, K. Pimenta, N.D. Goswami, A. Patrawalla, A. Lardizabal, C. Haley, L. Chen, L. Armitige, B. Seaworth, B. Sylvester, R. Bhavaraju

**Affiliations:** ^1^ Rutgers New Jersey Medical School, Newark, NJ, USA;; ^2^ School of Public Health, University of Memphis, Memphis, TN, USA;; ^3^ Division of Tuberculosis Elimination, Centers for Disease Control and Prevention, Atlanta, GA, USA;; ^4^ Rutgers Global Tuberculosis Institute, Newark, NJ, USA;; ^5^ Division of Infectious Diseases, Department of Medicine, Vanderbilt University Medical Center, Nashville, TN, USA;; ^6^ UCSF Curry International Tuberculosis Center, San Francisco, CA, USA;; ^7^ Heartland National TB Center, San Antonio, TX, USA;; ^8^ Division of Global Migration Health, Centers for Disease Control and Prevention, Atlanta, GA, USA.

**Keywords:** tuberculosis, medical consultation, guidance, immigration, panel physician

## Abstract

**BACKGROUND:**

Applicants seeking entry into the United States are examined overseas for TB by panel physicians and international immigration clinicians guided by Centers for Disease Control and Prevention (CDC) TB Technical Instructions. To support this effort, CDC-funded TB Centers of Excellence (COEs) provide web-based expert consultation, with documentation stored in a medical consultation database (MCD). MCD analysis can reveal inquiry trends among panel physicians worldwide.

**METHODS:**

TB-related queries in the COE MCD from January 1, 2018, to December 31, 2022, were analyzed using a descriptive coding scheme developed through inductive analysis, allowing multiple themes per entry.

**RESULTS:**

A total of 215 queries from 126 patients in 28 countries were analyzed. Major themes included evaluating diagnostic criteria, tailoring treatment, and managing comorbidities or adverse reactions. Diagnostic questions (*n* = 104, 48.4%) included mycobacterial culture, smear, and radiology interpretation. Treatment tailoring inquiries involved optimizing the initial regimen (*n* = 89, 41.4%) or modifying existing regimens (*n* = 26, 12.1%). Additionally, 50 consultations (23.2%) mentioned comorbidities, while 47 (21.9%) described adverse reactions.

**CONCLUSION:**

The MCD analysis identified topics where TB expertise was provided in overseas medical evaluation. These topics highlight opportunities for targeted panel physician education to improve the health of individual applicants and advance U.S. TB elimination efforts.

## RÉSUMÉ

**CONTEXTE:**

Les candidats souhaitant entrer aux États-Unis sont soumis à un examen à l'étranger pour détecter la TB, effectué par des médecins de groupe et des cliniciens spécialisés en immigration internationale, conformément aux directives techniques des Centers for Disease Control and Prevention (CDC) relatives à la TB. Pour appuyer cette initiative, les centres d'excellence sur la TB, financés par les CDC, offrent des consultations d'experts en ligne, dont les informations sont archivées dans une base de données de consultation médicale (MCD). L'analyse de cette base de données peut mettre en lumière les tendances des demandes d'information parmi les médecins du panel à l'échelle mondiale.

**MÉTHODES:**

Les demandes relatives à la TB dans la MCD du COE entre le 1er janvier 2018 et le 31 décembre 2022 ont été examinées à l'aide d'un système de codage descriptif élaboré par une analyse inductive, ce qui a permis d'identifier plusieurs thèmes par enregistrement.

**RÉSULTATS:**

Un total de 215 demandes émanant de 126 patients répartis sur 28 pays a été examiné. Les principaux thèmes abordés incluaient l'évaluation des critères diagnostiques, l'ajustement des traitements et la prise en charge des comorbidités ou des effets indésirables. Les interrogations liées au diagnostic (*n* = 104 ; 48,4%) concernaient la culture mycobactérienne, les frottis et l'interprétation des examens radiologiques. Les questions sur l'ajustement des traitements portaient sur l'optimisation du traitement initial (*n* = 89 ; 41,4%) ou sur la modification des traitements en cours (*n* = 26 ; 12,1%). De plus, 50 consultations (23,2%) faisaient état de comorbidités, tandis que 47 (21,9%) évoquaient des effets indésirables.

**CONCLUSION:**

L'analyse MCD a permis de déterminer les cas pour lesquels une expertise en TB a été apportée dans le cadre d'une évaluation médicale à l'international. Ces cas soulignent les opportunités de formation spécialisée pour les médecins du panel, dans le but d'améliorer la santé des patients concernés et de soutenir les initiatives d'éradication de la TB aux États-Unis.

TB is both preventable and curable,1 yet the WHO estimates that 10.6 million people fell ill with TB in 2021 alone,^1^ with one-third undiagnosed or untreated. Disparities in TB rates are striking, as incidence in low-income nations is more than 20 times that of high-income countries.2 Within high-income countries, TB prevalence is highest among migrants from countries with higher disease burden. Consequently, many countries have implemented strategies for TB evaluation among immigrating individuals.^2^

The U.S. CDC regulations require that persons applying for entry into the United States undergo a medical examination for health evaluation, including evaluation for TB^3,4^ These medical examinations are conducted by panel physicians, who are physicians practicing internationally to evaluate individuals applying for immigrant or refugee status, as well as non-immigrants who are required to have an overseas medical examination. Per the CDC, the number of persons examined by panel physicians globally (*N*) with the number of active cases of TB diagnosed (*n*) from 2018 to 2022 was follows: 2018 (*n*/*N*: 1,177/686,489); 2019 (*n*/*N*: 1,109/642,822); 2020 (*n*/*N*: 315/231,785); 2021 (*n*/*N*: 500/476,209); and 2022 (*n*/*N*: 700/657,679).

In brief, the TB screening process includes a medical history, physical exam, and possibly a chest X-ray. For persons with signs or symptoms of TB or with known HIV infection, the process also involves an interferon-gamma release assay (IGRA) or tuberculin skin test (TST), chest X-ray, and three sputum specimens for smears and culture plus molecular testing of the first specimen; for persons with positive cultures, drug susceptibility testing is performed. This schematic varies slightly between countries with low TB burden and high TB burden.

The CDC’s Division of Tuberculosis Elimination funds four TB Centers of Excellence for Training, Education, and Medical Consultation (TB COEs). From 2018 to 2022, the four TB COEs were the UCSF Curry International Tuberculosis Center (San Francisco, CA, USA); the Global Tuberculosis Institute at Rutgers, The State University of New Jersey (Newark, NJ, USA); the Heartland National Tuberculosis Center (San Antonio, TX, USA); and the Southeastern National Tuberculosis Center (Gainesville, FL, USA). The four COEs receive separate funding from the CDC Division of Global Migration and Quarantine (DGMQ) to guide panel physicians in screening and treating individuals for TB disease via a medical consultation database (MCD).^5,6^ Panel physicians are made aware of the consultation service by CDC-DGMQ’s regular communications. The MCD is a confidential, optional, web-based service wherein panel physicians can submit free-text written queries routed to expert clinicians at the four U.S. TB COEs for their review and written response.

The MCD is a free-response text system in which panel physicians are able to include any information they deem relevant, often including the patient’s age, medical and surgical history, medications, allergies, social history, if appropriate, review of systems, physical exam findings, recent lab results, relevant imaging, and, if applicable, any current or prior treatment for TB disease, as well as any limitations in screening or treating the patient (including information on medication availability locally, or other social or cultural factors affecting management). Therefore, the primary purpose of the queries submitted to the MCD is to receive expert guidance from U.S.-based physicians from the COEs regarding the treatment of individuals with TB

This study aims to analyze all the queries within the MCD, identify any recurrent trends in inquiries from panel physicians, and further elicit challenges during overseas TB evaluations. Characterizing COE consultations could be used to develop targeted training programs aimed at improving TB immigration screening and enhancing U.S. TB elimination efforts.

## METHODS

### Study design, data collection, inclusion criteria, and exclusion criteria

This retrospective analysis encompasses all panel physician queries in the COE MCD from January 1, 2018, to December 31, 2022. Sampling methods were not applicable to this study design, as no sampling was performed. Complete entries within this period were included. Duplicate entries were excluded. Entries that included only pleasantries without any patient or case information were excluded. This study was approved by the Rutgers University Institutional Review Board and did not meet the regulatory definition of human subjects research.

### Descriptive coding scheme

A descriptive coding scheme was developed through both deductive and inductive approaches. A deductive approach was first employed, in which initial themes were generated based on previously published analyses of domestic MCD consultations and existing panel physician guidelines.^7–9^ An inductive approach was next employed, in which three independent researchers reviewed an initial randomized subset of MCD queries to identify initial codes that captured the main themes of the inquiries. This comprised the qualitative analysis of the MCD queries in developing codes. The coding scheme was then collaboratively refined through discussion and agreement on the most applicable terminology. Final themes were then labeled as descriptive codes and used for data analysis. To maintain consistency, coding guidelines and procedures were developed to resolve discrepancies and reach consensus on codes for specific queries. The entries were cross-coded to ensure validity and reliability. There were no significant variations in the way two researchers independently blindly coded the same query. Minor discrepancies were resolved via open discussion among the research team.

### Data analysis

The finalized coding scheme was applied to all MCD queries using QualtricsXM^®^ (January 2023 version), a survey administration and analysis tool, using an approach in which multiple thematic codes could be applied per individual entry. Descriptive statistics were calculated in Qualtrics to quantify topic distribution. This comprised the quantitative analysis of data.

## RESULTS

**Figure 1.**
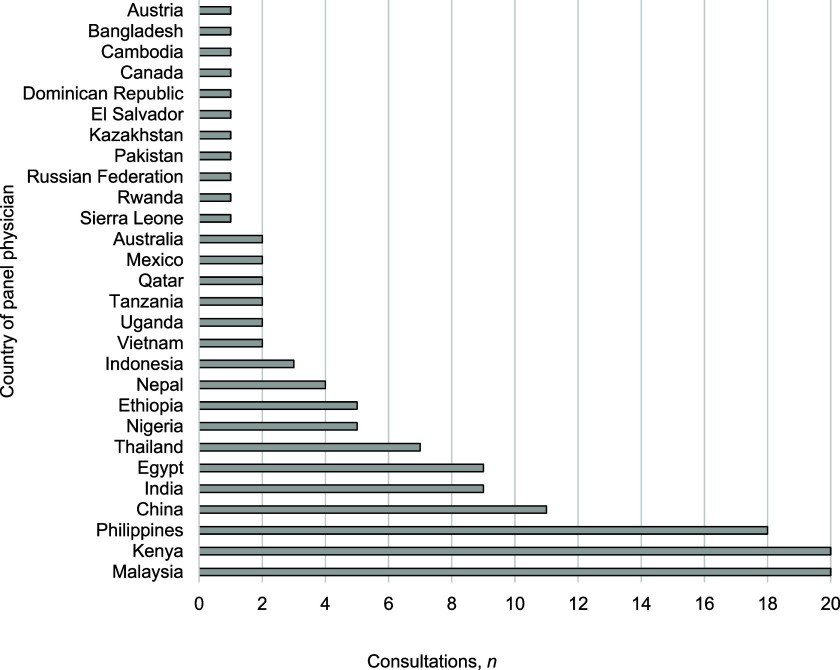
Panel physician consultations by country, 2018-2022.

**Figure 2.**
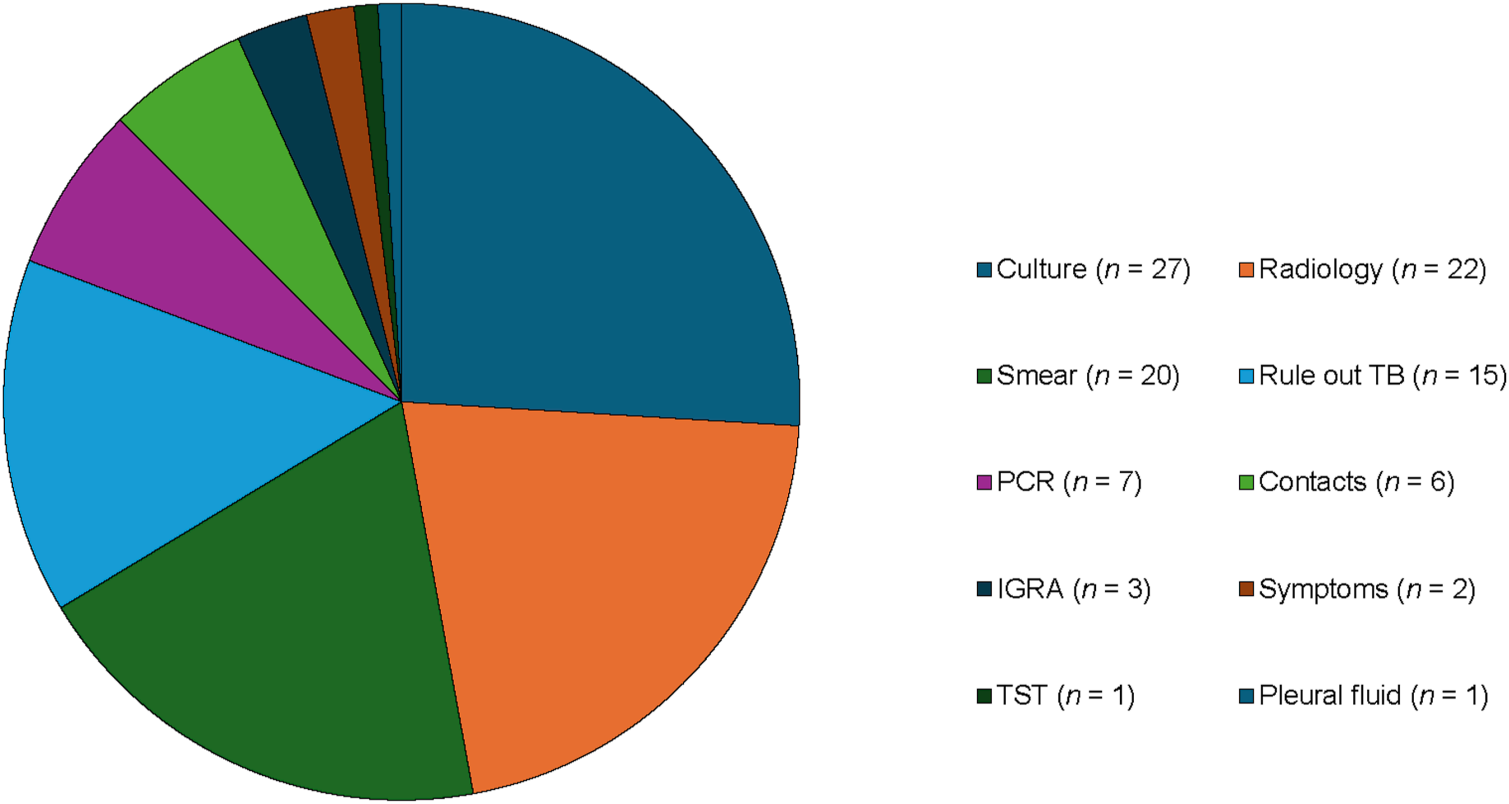
Number of consultations under the category ‘Diagnostics’: non-exclusive count of queries pertaining to diagnostic factors. PCR = polymerase chain reaction; TST = tuberculin skin test; IGRA = interferon-gamma release assay.

This analysis covered 215 queries (126 initial queries, 89 follow-ups) from panel physicians to consultant physicians at COEs from 126 TB patients representing 28 different countries. Geographically, panel physicians from Malaysia and Kenya submitted the highest volume of consultation inquiries, at 20 queries each, followed by panel physicians from the Philippines, with 18 queries (Figure 1). Major themes present in queries included TB diagnostic criteria, initial treatment selection, adverse reactions during treatment, treatment duration, and comorbidity management. TB diagnostic inquiries (*n* = 104, 48.4%), including interpretation of culture, smear microscopy, radiology, and other aspects involving the ascertainment of TB diagnoses, are presented in Figure 2.

Among all inquiries, 89 (41.4%) requested assistance selecting an optimal initial TB treatment regimen, while 26 (12.1%) were questioned about altering the baseline or initial treatment regimen, including transitioning to second-line regimens. Rationale for these regimen modifications included: availability of new drug susceptibility testing results (*n* = 17, 7.9%), treatment failure from prior regimen (*n* = 6, 2.8%), adverse reactions during TB treatment (*n* = 6, 2.8%), addition of antimycobacterial drugs as per updated evidence-based guidelines (*n* = 2, 0.9%), interruptions in treatment course (*n* = 1, 0.5%), drug formulations (1, 0.5%), and drug availability (*n* = 1, 0.5%).

Another common coding theme was treatment duration, involving 55 (25.6%) inquiries. Panel physicians sought guidance on the optimal duration of treatment using first- or second-line antimycobacterial agents. COE consultants considered factors including disease severity, potency of antimycobacterial drugs included in the treatment regimen, time-to-culture-conversion as a measure of mycobacterial burden, clinical and radiographic improvement, medication tolerability, TB-isolate drug resistance profiles, and other individual patient characteristics.

Comorbidities were mentioned in 50 (23.2%) of the consultations. Most frequently cited comorbidities included diabetes (*n* = 9, 4.2%), liver disease (*n* = 9, 4.2%), hypertension (*n* = 8, 3.7%), depression (*n* = 6, 2.8%), and HIV (*n* = 3, 1.4%). Adverse reactions to TB treatment were described in 47 (21.9%) inquiries, with gastrointestinal (*n* = 10, 4.7%) and hepatotoxic (*n* = 8, 3.7%) side effects being the most common.

## DISCUSSION

Analysis of the MCD revealed areas requiring expert guidance in managing TB during overseas panel physician evaluations, including TB diagnostic testing, initial treatment selection or treatment modifications from baseline, treatment duration, and management of comorbidities and adverse drug reactions. Identifying these trends in queries by panel physicians allows for targeted training programs and educational resources. Developing focused interventions may better equip panel physicians to manage patients with complex TB, improve physician competency in critical areas, and enhance their ability to provide comprehensive TB evaluations for patients seeking immigration to the United States.

This analysis revealed geographical variations in the volume of consultations received and addressed by the COEs. The utilization of the MCD service by panel physicians in Malaysia, Kenya, and the Philippines may reflect an increased awareness among panel physicians of such a resource for clinical decision-making and guidance. However, the geographic difference in consultation volume may also represent an opportunity to address the specific needs of panel physicians in these regions via a dedicated subgroup analysis to identify trends in queries and could, therefore, allow for the optimization of educational resources to meet the needs of panel physicians in these specific countries.

Several questions in the MCD pertained to the diagnosis of TB, including interpretation of culture, sputum smears, and radiological interpretation. Many of these diagnostic inquiries involved specific circumstances, including lapses in treatment or continuity of care, particularly concerning CDC TB Classification and Travel Clearance status. These queries reflect the dual role of panel physicians in navigating the immigration landscape and providing high-quality care to those seeking immigration. This, therefore, may reflect a need to clarify or provide additional guidance in assigning TB classification status.

A high volume of queries in the MCD involved the duration of treatment, often related to highly complex medication regimens. Decisions regarding extending treatment duration require nuanced clinical judgment, and the consultant physicians at the COEs are well-positioned to continue providing expertise in this area. The volume of queries relating to the duration of treatment reflects an appropriate use of the MCD service, indicating that the MCD is being utilized to provide expert guidance, including evidence-based updates and treatment tailoring specific to individual patients with highly complex TB Additionally, this analysis identified a high volume of queries relating to the management of comorbidities and adverse reactions to TB treatment. These queries reflect the importance of a holistic approach to patient care and the management of TB Panel physicians may require further training regarding the management of special populations of patients with TB, including patients with coexisting diabetes, liver disease, and hypertension. A thorough understanding of these comorbidities may allow panel physicians to manage better bidirectional interactions between antimycobacterial medications and medications for comorbid medical conditions.

Moreover, providing care to patients with TB who may be otherwise medically complex requires a careful understanding of further intensive interventions, such as nutritional support or evaluation of malabsorption. Receiving targeted training in these areas may allow panel physicians to optimize patient outcomes, ensure patient safety, and encourage medication adherence. Based on the themes present in the inquiries, tailored educational material can be developed to train panel physicians on these topics specifically. Of note, clinicians from the U.S. TB COEs have in the past visited panel physicians abroad to conduct education and training, most recently in 2022 to Kenya, Nigeria, and Niger. During future similar visits, COE clinicians may use insights gathered from this analysis to provide tailored education on topics that appear most frequently in the panel physician queries.

Further efforts may be made to conduct follow-up interviews with panel physicians to help identify changes in practice and training gaps. In summary, insights from this analysis could advance U.S. TB elimination efforts by improving overseas TB evaluations and preventive measures. Targeted training and education for panel physicians in the areas identified by this study could improve evaluation accuracy, quality, and patient outcomes.

### Limitations

There are several limitations of this retrospective analysis. Given that the study is limited only to the COE MCD dataset, it is possible that not all consultations or relevant queries were captured. While TB COE consultation services are available to all panel physicians, not all may be aware of the service, despite CDC efforts to increase awareness and use of this resource among panel physicians. Some panel physicians may use this COE MCD service more than others, and these clinicians are better represented in the MCD. Moreover, as the data were collected from 2018 to 2022, the analysis might not be generalizable to other time periods, and the volume or quality of consults may have been affected by the COVID-19 pandemic and the impact of the pandemic on U.S. immigration. The challenge of generalizing MCD data to the broader population restricts external validity, thereby impeding global efforts towards TB eradication. Additionally, the analysis focused only on inquiries made to TB COEs and did not capture queries addressed by other sources (e.g., published guidelines, experienced colleagues, CDC Quality Assessment Program resources). Lastly, coding decisions involve some subjectivity, though significant efforts were made to minimize bias.

## CONCLUSION

This analysis highlights areas where TB expertise is required during overseas medical evaluations. Targeted training can enhance panel physicians’ competence, improve overseas TB evaluations, and contribute to U.S. TB elimination efforts. These findings can inform policy decisions, resource allocation, and support systems for panel physicians, ultimately improving immigrant health.

## References

[1] World Health Organization. Global tuberculosis report, 2022. Geneva, Switzerland: WHO, 2022.

[2] Pescarini JM, Migration to middle-income countries and tuberculosis: global policies for global economies. Glob Health. 2017; 13:15. 10.1186/s12992-017-0236-6 PMC5353961 28298223

[3] Posey DL, Implementation of new TB screening requirements for U.S.-bound immigrants and refugees, 2007–2014. MMWR Morb Mortal Wkly Rep. 2014;63(11):234–236. 24647399 PMC4584633

[4] U.S. Department of Health & Human Services, Centers for Disease Control and Prevention. Tuberculosis technical instructions for panel physicians. Atlanta, GA, USA: CDC, 2019.

[5] U.S. Department of Health & Human Services, Centers for Disease Control and Prevention. TB Centers of Excellence for training, education, and medical consultation. Atlanta, GA, USA: CDC, 2018.

[6] U.S. Department of Health & Human Services, Centers for Disease Control and Prevention. TB medical consultation service. Atlanta, GA, USA: CDC, 2019.

[7] Gobaud AN, Multidrug-resistant tuberculosis care in the United States. Int J Tuberc Lung Dis. 2020;24(4):409–413. 32317065 10.5588/ijtld.19.0515 PMC9325499

[8] McDowell A, Linezolid use for the treatment of multidrug-resistant tuberculosis: TB Centers of Excellence, United States, 2013–2018. J Clin Tuberc Other Mycobact Dis. 2020; 22:100201. 33336084 10.1016/j.jctube.2020.100201 PMC7732868

[9] Fernando R, A model for bringing TB expertise to HIV providers: medical consultations to the CDC-funded regional tuberculosis training and medical consultation centers, 2013–2017. PLoS One. 2020;15(8):e0236933.0. 32866154 10.1371/journal.pone.0236933 PMC7458296

